# Humic Substances Derived From Biomass Waste During Aerobic Composting and Hydrothermal Treatment: A Review

**DOI:** 10.3389/fbioe.2022.878686

**Published:** 2022-05-12

**Authors:** Zhong-Ting Hu, Weizhong Huo, Yue Chen, Qiang Zhang, Mian Hu, Weicheng Zheng, Yuchao Shao, Zhiyan Pan, Xiaonian Li, Jun Zhao

**Affiliations:** ^1^ College of Environment, Zhejiang University of Technology (ZJUT), Hangzhou, China; ^2^ Industrial Catalysis Institute, Zhejiang University of Technology, Hangzhou, China; ^3^ School of Environment, Tsinghua University, Beijing, China; ^4^ Hangzhou Guotai Environmental Protection Technology Co. LTD, Hangzhou, China; ^5^ Hangzhou Research Institute of China Coal Technology & Engineering Group, Hangzhou, China; ^6^ Department of Biology, Institute of Bioresource and Agriculture, Hong Kong Baptist University, Kowloon Tong, Hong Kong SAR, China

**Keywords:** biomass waste, humic substances, aerobic composting, hydrothermal treatment, humification

## Abstract

Humic substances (HSs) occupy 80% of organic matter in soil and have been widely applied for soil remediation agents, potential battery materials, and adsorbents. Since the HS extraction rate is very low by microbial degradation in nature, artificial humification processes such as aerobic composting (AC) and hydrothermal treatment (HT) have attracted a great deal of attention as the most important strategies in HS production. This article aims to provide a state-of-the-art review on the development of conversion of biomass waste into HSs based on AC and HT for the first time in terms of mechanisms, characteristics of HSs’ molecular structure, and influencing factors. In addition, some differences based on the aforementioned information between AC and HT are reviewed and discussed in the conversion of biomass waste into HSs in a pioneering way. For biomass waste conversion, a feasible strategy on effective humification processes by combining AC with HT is proposed.

## 1 Introduction

Humic substances (HSs) significantly influence soil physicochemical properties because the organic matter in soil comprises over 80% HSs (Shan et al., 2010). As defined by the International Humic Substance Society (IHSS), HSs are derived from dead plants and microbial remains by humification after undergoing a series of physical effects and chemical reactions in nature (Hayes & Swift, 2020). The precursors could be lignin, polysaccharide, melanin, cutin, protein, lipid, nucleic acid, and fine char particle, etc. HSs have been widely used as (1) a soil remediation agent in agriculture, (2) a kind of absorbent for removing organic pollutants and heavy metals, (3) a kind of potential battery material due to its high storage capacity, and (4) a type of additive in elastic concrete (Yang & Antonietti, 2020). For instance, the group tested the adsorption of humic acid (HA) for cadmium and indicated that HA can effectively prevent the adsorption of Cd by soil (Zheng et al., 2022). Some researchers compounded HA with Fe@Fe_2_O_3_ to degrade 2,4,6-trichlorophenol (Zhang et al., 2022). The degradation efficiency of 2,4,6-trichlorophenol in this catalytic system was improved from 59% to 83% due to the enhancement of peroxymonosulfate activation and abundant functional groups. Although HSs present great potential for applications, their composition is extremely complex with the formation of the chemical structure randomly undergoing slow microbial degradation in nature. In general, HSs can be divided into HA, fulvic acids (FAs), and humins based on their characteristics in alkaline and acidic solutions.

**FIGURE 1:**
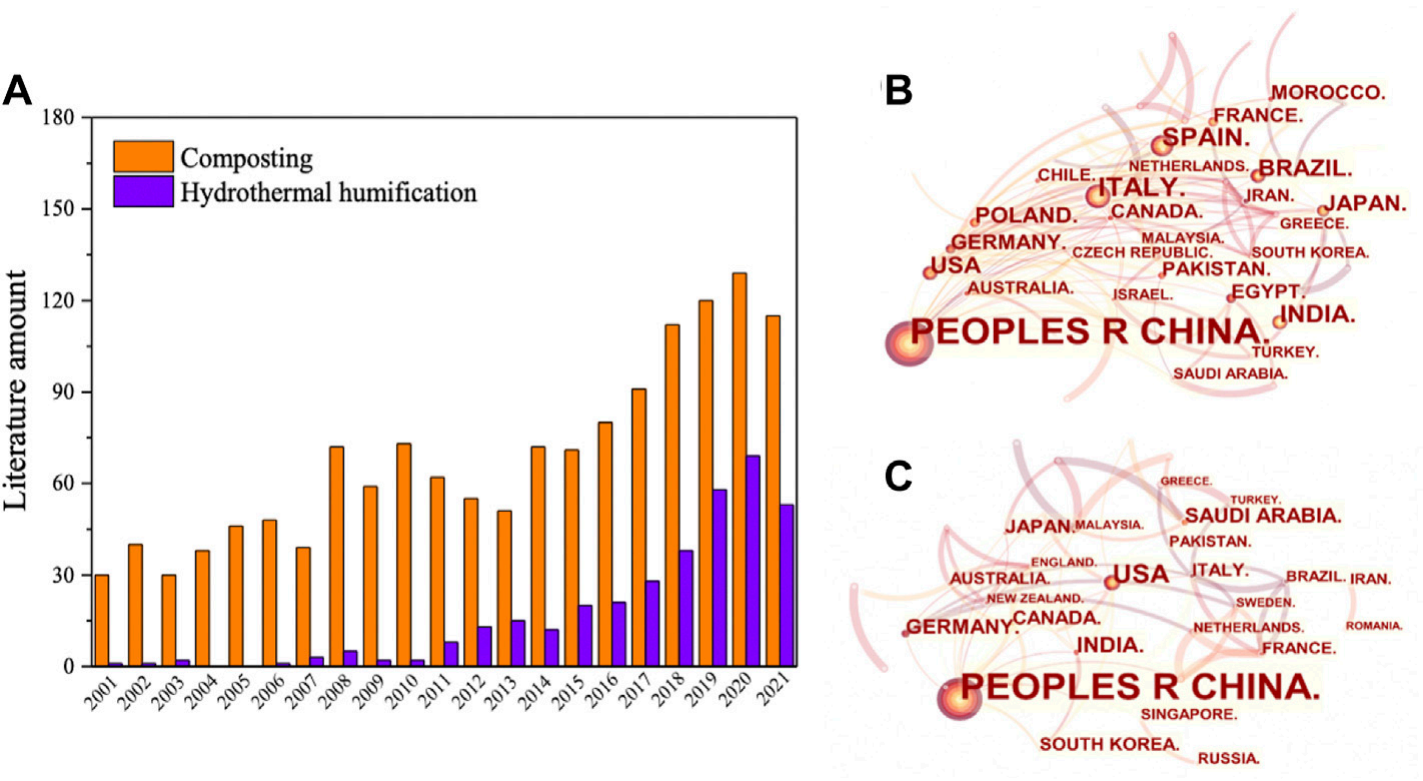
Recent studies on composting and hydrothermal treatment for biomass humification. **(A)** Amount of relative literatures from 2001 to 2021; **(B)** research interest in the field of composting humification in major countries; **(C)** research interest in the field of hydrothermal humification in major countries.

Considering the much slower rate of biomass humification in the natural soil environment, for example, HS formation at a soil depth of more than 16 m needs over 15,000 years (Visser, 1962), and it is necessary to develop artificial techniques in humification processes. At present, the mainstream technologies for humification processes of biomass waste are aerobic composting (AC) and hydrothermal treatment (HT) (Adani et al., 1999; Du & Li, 2017; Liu et al., 2019; Yang et al., 2019). AC, a kind of biochemical process, can be used to convert biomass in municipal solid waste to stable HSs under controlled conditions, in which the microorganisms could be bacteria, actinomycetes, and fungi that existed widely in nature (Guo et al., 2019; Ding et al., 2021). In contrast, HT can rapidly transform biomass waste into value-added products such as hydrochar (it is also called humins in some cases), chemicals, biogases, and HSs in hours or minutes (Kruse et al., 2013; Kang et al., 2018; Yang et al., 2019; Yang D. et al., 2020; Zhu X. et al., 2020; Ma et al., 2020; Shao et al., 2020; Li et al., 2021; Meng et al., 2021; Ni et al., 2021). Until now, 1682 articles published on the topic of “composting for humification conversion from biomass” have been found by searching keywords of “composting and humic substances” or “composting and humic acid” or “composting and fulvic acid” or “composting and humin” from all databases. There are 365 articles based on HT in this field. It is worth noting that the number of published articles in recent times regarding the humification effect of biomass waste is increasing, especially in China (Figure 1).

In those published articles, AC integrated with HT for biomass conversion has been considered a new promising method (Nakhshiniev et al., 2014; Nakasaki et al., 2015; Gong et al., 2021). In this system, HT, a pre-treatment process, can efficiently transform non-biodegradable biomass waste (e.g., cellulose and lignin) into small organic molecules followed by enhancing humification processes in AC. To the best of our knowledge, there is no overview on the comprehensive summarization of humification processes of biomass waste based on composting integrated with HT processes.

This study aims to provide a timely review on the development of humification processes of biomass waste based on AC and HT. The topics discussed include a brief summary of the physicochemical characteristics of humic substances (HSs) and mechanisms and influencing factors within individual humification processes. The challenges and prospects of integrating AC with HT for an advanced humification process of biomass waste conversion are presented by reviewing individual features and their differences.

## 2 Physicochemical Characteristics of Humic Substances

Physicochemical characteristics of humic substances (HSs), which are related to molecular structure’s main influence, are the main research focus in humification processes (Beyer, 1996; Hayes et al., 2017; Hayes & Swift, 2020). The standard detection techniques, including Fourier transform infrared spectroscopy (FI-IR), nuclear magnetic resonance (NMR), excitation–emission matrix spectroscopy (EEM), and elemental analysis, have been applied for investigating the physicochemical characteristics of HSs (Chukov et al., 2018; Enev et al., 2014; Fuentes et al., 2018; Hu et al., 2018; Hu et al., 2021; Jacquin et al., 2017). As shown in Table 1, the major FT-IR absorption bands and chemical shifts of C^13^ atoms in HSs indicate abundant O-containing, aromatic, and/or N-containing structures in HS molecular structure. The EEM spectra, a typical technology, can evaluate the HS content in the liquid phase. It has been analyzed in detail that the spectrum can be separated into four parts involving aromatic protein–like fluorophores, fulvic acid–like fluorophores, soluble microbial product–like fluorophores, and humic acid–like fluorophores (Jacquin et al., 2017). Therefore, this analytical method can extremely quantify the HA and FA content in HSs. Researchers also detected the element composition of the HSs to investigate the carbon, hydrogen, and oxygen distribution (Sumerskii et al., 2010; Kang et al., 2016; Fuentes et al., 2018; Ding et al., 2021). The representative examples have been presented in Table 2. HSs have different carbon content ranging from 39% to 75% according to the products of the standard authorized by IHSS, i.e., those are available commercially in Aldrich, those extracted from biomass waste, and those prepared by hydrothermal humification, indicating that the chemical structure of HSs is very complex.

**TABLE 1:** Major Fourier transform infrared spectroscopy (FT-IR) absorption bands and chemical shifts of C^13^ atoms in molecular fragments of humic substances (Enev et al., 2014; Chukov et al., 2018).

FT-IR wavenumber (cm^−1^)	Assignment
3400–3300	O–H stretching, N–H stretching (minor), and hydrogen-bonded OH
2935–2925 and 2850	asymmetric and symmetric C–H stretching of the CH_2_ group
1725–1710	C=O stretching of COOH
1640–1600	aromatic C=C skeletal vibrations, C=O stretching of amide groups (amide I band), and C=O of quinone and/or H-bonded conjugated ketones
1512–1506	N–H deformation and C=N stretching (amide II band) and aromatic C=C stretching
1460–1450	C–H asymmetric bending of CH_3_ groups
1420–1415	O–H deformation and C–O stretching of phenolic OH
1380	C–H bending of CH_2_ and CH_3_ groups and COO^−^ anti-symmetric stretching
1270–1260	C–O stretching of aryl esters
1220	C–O stretching of aryl ethers and phenols
1184	C–O–C stretching (skeletal vibration) of cellulose residues
1130–1110	C–O stretching of secondary alcohols and/or ethers
1045–1035	C–O stretching of polysaccharides or polysaccharide-like substances and/or Si-O of silicate impurities
660–620	S–O stretching vibration sulfonic groups
**Chemical shift, ppm**	**Assignment**
** **0–47	C- and H-replaced aliphatic fragments
** **47–60	Methoxyl and O- and N-replaced aliphatic fragments
** **60–108	Aliphatic fragments double replaced by heteroatoms (including carbohydrate) and methylene carbon of ethers and esters
** **108–144	C- and H-replaced aromatic fragments
** **144–164	O- and N-replaced aromatic fragments
** **164–183	Carboxyl groups, amides, and their derivatives
** **183–190	Quinone groups
** **190–204	Aldehyde and ketone groups

**TABLE 2:** The element composition (w/w%) of the HSs.

Sample	C	H	O	N	References
LSHA	63.8	3.7	31.3	1.2	Fuentes et al. (2018)
PRHA	56.4	3.8	37.3	3.7	
SRFA	53	4.4	43.9	0.8	
WRFA	53.6	4.2	41.8	1.1	
ESFA	49.79	4.27	44.34	3.25	
PSFA	51.31	3.53	43.32	2.34	
NRFA	52.31	3.98	45.12	0.68	
AHA	60.4	4.4	34.5	0.7	
HS1	59.5	3.5	36.3	0.6	
HS2	74.2	2.8	21.9	1.2	
HS3	64.7	4.2	30	1.1	
HS4	57.6	7.0	34.2	1.3	
HS5	65.3	3.7	29.6	1.4	
apHS1	51.1	5.4	39.7	3.8	
apHS2	39.2	4.2	56.7	0	
apHS3	48	5.5	44.1	2.4	
apHS4	59.2	5.6	33.1	2.1	
apHS5	59.6	5.3	35.2	0	
apHS6	62.7	5.5	32.1	0	
apHS7	56.5	4.5	38.4	0.6	
apHS8	56.7	7.6	19.0	16.6	
apHS9	44.7	5.1	36.7	13.5	
apHS10	47.3	4.5	44.6	3.5	
Fructose derived humins	62.08	4.39	33.22	0.31	Shao et al. (2021)
	62.29	3.88	33.63	0.20	
	62.08	4.03	33.71	0.18	
	62.96	4.43	32.56	0.05	
	62.75	4.42	32.79	0.04	
	63.31	4.44	32.18	0.07	
Glucose derived humins	66.4	4.7	28.9	/	Sumerskii et al. (2010)
	58.2	4.5	37.3	/	Kang et al. (2016)

Note: LSHA, Leonardite Standard Humic Acid; PRHA, Pahokee Peat Reference Humic Acid; SRFA, Suwanne River Reference Fulvic Acid; WRFA, Waskish Peat Reference Fulvic Acid; ESFA, Elliot Soil Standard Fulvic Acid; PSFA, Pahokee Peat Standard Fulvic Acid; NRFA, Nordic Lake Reference Fulvic Acid; AHA, a commercial humic acid obtained from Aldrich Chemicals; HS1, humic substances extracted from humified materials: a ; HS2,leonardite from Florida a Chinese leonardite; HS3, a Czech leonardite; HS4, a Spanish peat; HS5, a Russian lignite; the apparent humic substances (apHS) were extracted from seven lignosulfonates (apHS1–apHS7), two protein-derived products, consisting of a pool of oligopeptides and amino acids (apHS8 and apHS9) and an extract from seaweed (apHS10).

Fructose derived humins and glucose derived humins obtained from hydrothermal reaction.

**FIGURE 2:**
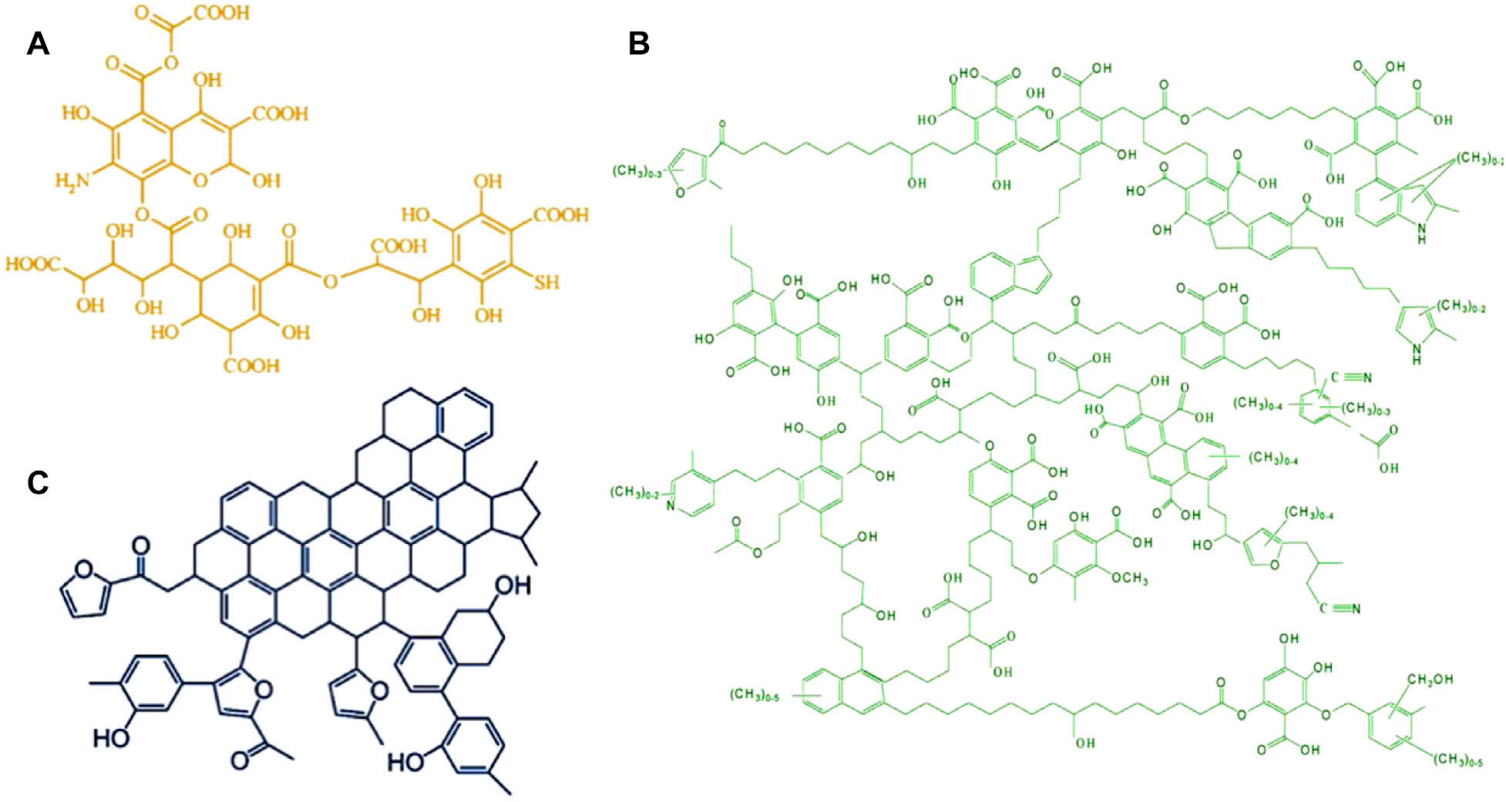
Proposed structure models of fulvic acid **(A)** reproduced from the work by Alvarez-Puebla et al. (2006) with permission from Elsevier (copyright 2006), humic acid **(B)** reproduced from the work by Terkhi et al. (2008) with permission from Elsevier (copyright 2008), and humins **(C)** reproduced from the work by Chuntanapum and Matsumura (2009) with permission from ACS Publications (copyright 2009).

Pyrolysis gas chromatography/mass spectrometry (Py-GC/MS) can also be used to investigate HSs’ chemical properties. Buurman et al. (2009)applied the Py-GC/MS to reveal the source of five HA samples from different EUROSOILS. The result showed that the HA from different sources has different component groups (i.e*.*, carbohydrate, protein, lignin, lipid, carbonyl, and char) after pyrolysis was analyzed by applying the molecular mixing model, indicating the complexity of HS formation again. Fuse et al. (2016) analyzed the typical pyrolytic gas for five HA samples extracted from different sedimentary layers. The same results can reflect that the five HA samples containing polyphenols, lignin, proteins, amino acids, polysaccharides, and lipids are different. Pyrolysis of humins from carbohydrates produces complex components, including furanics, lactones, phenolics, and organic acids (Rasrendra et al., 2013). Therefore, a deep investigation on the representative molecule structures of HSs is needed. The typical models of HSs (FA, HA, and humins) with different structures were provided by the research studies (Alvarez-Puebla et al., 2006; Terkhi et al., 2008; Chuntanapum & Matsumura, 2009), which have been used till now (Figure 2).

## 3 Biomass Waste Humification Processes

### 3.1 Conversion Pathway of Biomass Waste Into HSs

There are several plausible deductions on the formation pathways of HSs based on varied processes of lignin oxidation, lignin polyphenol polymerization, microbial polyphenol synthesis, cellular autolysis, sugar–amine condensation, coalification, and anaerobic fermentation (Guo et al., 2018). In this review article, AC and HT applied for HS formation were introduced later.

**FIGURE 3:**
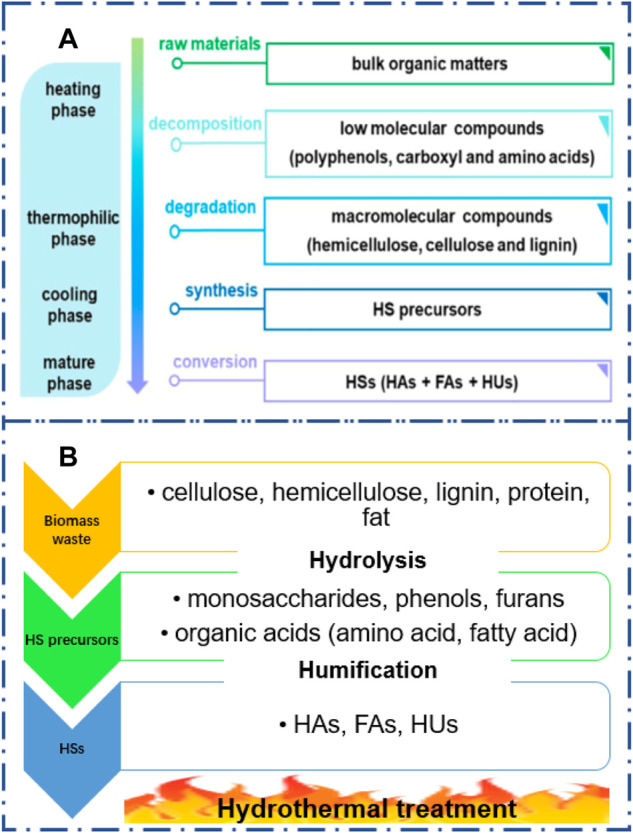
Proposed mechanisms of humic substance formation during aerobic composting **(A)** and hydrothermal reaction **(B)**. **(A)** Reproduced from the work by Guo et al. (2019) with permission from Elsevier, copyright 2019.

The mechanism of humification of biomass waste by AC is presented in Figure 3A, referring to the literature (Guo et al., 2019). Briefly, the easily degradable matter including the low molecular components such as polyphenols, carboxyl, and amino acids are decomposed due to the microorganism activity during the heating phase; then, the macromolecular compounds such as cellulose, hemicellulose, and lignin begin to degrade during the thermophilic phase; the large amount of HS precursors are produced in the whole cool phase, and subsequently they are converted into HSs during the mature phase. It was reported that lignin with aromatic structures can easily interact with the other compounds such as proteins, amino acids, and nucleic acids which is beneficial for HS formation (Sánchez–Monedero et al., 1999).

The HS formation can be accelerated in HT of biomass waste (Figure 3B). The biomass waste with abundant cellulose, hemicellulose, lignins, proteins, and lipids will be rapidly hydrolyzed into small molecules such as monosaccharides, phenols, furans, and organic acids in a short reaction time (Marzbali et al., 2021). Subsequently, these chemicals will be polymerized into HSs *via* Maillard reaction and/or aldol reaction (Zhu C. et al., 2020; Shen et al., 2020). It was found that the pH value can significantly affect the chemical composition during biomass degradation in hydrothermal reactions. For example, glucose under acidic hydrothermal conditions prefers to transfer into furans due to dehydration (Shao et al., 2021). In contrast, lactic acid, formic acid, and acetic acid can be achieved under alkaline hydrothermal conditions due to the reverse aldol condensation (Gao et al., 2013). These chemicals are all the potential precursors for HS formation. Further studies need to reveal the mechanism of HS formation during hydrothermal reaction.

### 3.2 Aerobic Composting for Biomass Waste Humification

HSs are a vital indicator to evaluate the quality of compost products. The factors influencing HS formation are complex, so it is of great significance to study the humification of biomass waste during AC. This section first summarized recent literatures about the effect of various parameters on the degree of humification *via* composting.

1) Ambient temperature

During AC, the complex biochemical reactions can cause an autothermal effect on the pile (Putranto & Chen, 2017). However, the ambient temperature can also affect the progress of pile humification. Low ambient temperature usually reduces the heating rate of the composting pile and shortens the time of the thermophilic phase, not conductive to the pile maturity. Chang et al. (2017) set the ambient temperatures of two piles at 10°C (CT10) and 25°C (CT25) to investigate the influence of this parameter on pile maturity (Table 3, entry 1). The CT25 case has a higher heating rate at the beginning of the composting period and also has a longer thermophilic period than CT10, indicating the great effect of ambient temperature on biomass composting. The HA content in piles, a key indicator reflecting the degree of pile maturity, increased during the composting period. The HA content of the CT25 treatment reached 24.18% at the end of composting, which was significantly higher than that of the CT10 treatment (5.52%). In addition, CT25 treatment achieved maturity quicker, indicating that a higher ambient temperature is beneficial for pile humification. A similar conclusion can be found in other literatures which indicated that the composting in summer reached the mature phase quicker than that in winter (Tiquia et al., 1997). However, a high ambient temperature is harmful for composting due to the inhibition of microbial activities at the initial stage. It was reported that a suitable ambient temperature of the composting pile is located at 15–45°C (Bhatia et al., 2013). Different composting substances need their own suitable ambient temperature, but most composting processes often overlook this effect.

2) Raw material type

**TABLE 3:** Effects of various parameters on the degree of humification during composting.

Entry	Substance	Variable		Other condition	Degree of humification	Reference
1	Dewatered sludge + wheat straw (1:1)	Ambient temperature (°C)	10 (CT10)	Moisture content: 60%; aeration rate: 60 ml/min; composting period: 30 days	The HA content tended to increase during the whole period, and at the end of the composting period, the HA content of the CT25 treatment reached 24.18%, which was 5.52% higher than that of the CT10 treatment	Chang et al. (2017)
			25 (CT25)			
2	Biomass	Raw material type	Willow and hay biomass (WBC)	Composting period: 167 days for WBC, 180 days for MSWC, and 149 days for MSWC2	The HA yield for the WBC was low only in the range of 4.3–8.5%, while the HA yields for the MSWC and MSWC2 were 14.0–47.5% and 4.2–14.7% of TOC	Kaluza-Haladyn et al. (2019)
			Unsorted municipal waste in the Katowice (MSWC)			
			Municipal waste from Zielona Góra (MSWC2)			
3	Biomass	Raw material type	Cow dung + maize straw (T1)	Moisture content: 60%; C/N ratio: 27; composting period: 35 days	The total HA content of T3 and T4 was significantly higher than that of T1 and T2	Li et al. (2019)
			Cow dung + wheat straw (T2)			
			Sheep dung + maize straw (T3)			
			Sheep dung + wheat straw (T4)			
4	Pig manure + corn straw	C/N ratio	15	Composting period: 120 days; aeration rate: 0.16 L kgDM^−1^ min^−1^	The FT-IR results indicated that the HA obtained at a C/N ratio of 15 has higher aromatization and stable structure	Sun et al. (2014)
			25			
			35			
5	Cow dung + corn straw	C/N ratio	15 (R1)	Composting period: 45 days; moisture content: 65%	Compared with 0 days, the increase of HA content in T4 and T5 treatments was 23.5–33.1% lower than that in T1, T2, and T3 treatments	Yin et al. (2019)
			20 (R2)			
			25 (R3)			
			30 (R4)			
			35 (R5)			
6	Chicken manures + corn straw	Additives	Control group (CK)	Composting period: 60 days	The FA content decreased from 50.73 g/kg to 37.85 g/kg and 36.38 g/kg to 23.56 g/kg in CK and MnO_2_ treatments, respectively; the HA yields were 13.24% in the CK group and 21.39% in the MnO_2_ group	Zhang et al. (2020)
			MnO_2_	Moisture content: 55–65%; C/N ratio: 20		
7	Chicken manures	Additives	CK	Composting period: 49 days	The HA yield first decreased and then increased during composting, and the HA yields in ATP and MA treatments were higher than those in the control group	Wang et al. (2019)
			Adenosine triphosphate (ATP)	Moisture content: 50–60%; C/N ratio: 30; the ventilation rate: 0.5 L min^−1^ kg^−1^		
	Garden waste		Malonic acid (MA)			
8	Pig manure + sawdust	Additives	0% medical stone (MS)	Composting period: 60 days	The FA content was degraded in relation to the increase of MS amount. At the end of composting, with the increase in the MS amount, the HA content was increased to 15.13, 16.36, 16.95, 17.90, and 18.81%	Wang et al. (2017b)
			2.5% MS	Moisture content: 55–60%		
			5.0% MS			
			7.5% MS			
			10.0% MS			
9	Maize straw + canola residue	Additives	No inoculation (S1)	Composting period: 36 days; moisture content: 52%; C/N ratio: 25	Compared with that of T1 and T3, the HA content of T2 treatment increased rapidly from 18 days, and by the end of composting, it was significantly higher than that in T1 and T3, reaching 94.97 g kg^−1^	Chen et al. (2019)
			Inoculation of *P. chrysosporium* on day 18 (S2)			
			Inoculation of *P. chrysosporium* on day 0 (S3)			
10	Rice straw, vegetables, bran, and soil (11:3:2:8)	Additives	No inoculation (Run 1)	Composting period: 42 days; moisture content: 55%; C/N ratio: 30	The humification ratio first increased and then decreased in Run 1 and 2, while it increased during the whole composting in Run 3, and the highest value of 51.25 was obtained	Zeng et al. (2009)
			Inoculation of *P. chrysosporium* on day 2 (Run 2)			
			Inoculation of *P. chrysosporium* on day 15 (Run 3)			
11	Kitchen waste + garden waste (17:3)	Aeration intensity (L kg^−1^ DM min^−1^)	0.24	Composting period: 35 days; moisture content: 66.8%	Low aeration intensity could enhance the molecular weight and polymerization of humic substances	Xu et al. (2021)
			0.36			
			0.48			
12	Digestates + chicken manure	Aeration intensity (L kg^−1^ DM min^−1^)	0.05	Composting period: 60 days; C/N ratio: 27.6	The HS concentration was increased by 21.1, 26.4, and 22.4% when the aeration rates were 0.05, 0.1, and 0.15 L kg^−1^ DM min^−1^, respectively	Wu et al. (2019)
			0.1			
			0.15			
13	Dairy manure (100 kg)	Pretreatment method	Thermal pretreatment composting (TPC)	Composting period: 60 days; moisture content: 75.1%	Thermal pretreatment significantly enhanced the humification degree of composting	Zhu et al. (2021)
			Traditional composting (TC)			
14	Chicken manure + straw	Pretreatment method	One-time composting fermentation	Composting period: 10 days; C/N ratio: 25	HS and HA content increased by 3.3%–32.1% and 1.7%–56.1% under the secondary fermentation, compared to other treatments	Luan et al. (2020)
			Continuous-composting-fermentation			

Raw materials for AC generally contain livestock and poultry manure, urban sludge, kitchen waste, and straw waste. The variety and complexity of feedstock refer to the different physicochemical characteristics of the composting piles, indicating that the type of raw materials for composting is an important parameter in humification processes. Kaluza-Haladyn et al. (2019) chose the willow and hay biomass (WBC) and unsorted municipal waste in the Katowice agglomeration (MSWC) and municipal waste from Zielona Góra (MSWC2) as feedstock for composting, aiming to assess the HS transformation during composting. The HA yield for the WBC was low only in the range of 4.3–8.5%, while the HA yields for the MSWC and MSWC2 were 14.0–47.5% and 4.2–14.7%, respectively, indicating that different types of raw materials notably affect the degree of humification in composting. Li et al. (2019) tested four kinds of feedstock for composting, as shown in Table 3 (entry 3). The similar result also observed that the total HA content of T3 (sheep dung + maize straw) and T4 (sheep dung + wheat straw) was significantly higher than that of T1 (cow dung + maize straw) and T2 (cow dung + wheat straw) during the whole composting period, ascribing to the higher content of cellulose and hemicellulose in T3 and T4, which is beneficial for HS formation. Therefore, the influence of the proportion of compounds in the composting pile on humification needs to be concerned. It is suggested to classify the main components of the pile and explore the influence of each component including trace elements such as metal ions on compost humification. A correlation model between the content of each compound and humification processes during composting can be established to provide a reference database for the selection of raw material ratios to achieve rapid and stable humification more effectively.

3) C/N ratio

The C/N ratio greatly related with the types of raw materials has further attracted attention because it significantly affected humification processes in AC. Sun et al. (2014) set 3 C/N ratios of composting piles by adjusting the proportion of pig manure and corn straw as shown in Table 3 (entry 4). The HA termly sampled from each pile was analyzed by FT-IR, and the result showed that the HA obtained at a C/N ratio of 15 has a higher aromatization and stable structure. HA has more polysaccharides and fatty components at a C/N ratio of 25, while there are less polysaccharides and more fatty components contained in HA at a C/N ratio of 35. It indicates that the C/N ratio greatly affects the HA structure. In term of HS yield influenced by the C/N ratio, there are also some research studies focused on it. Yin et al. (2019) tested 5 C/N ratios (R1 = 15, R2 = 20, R3 = 25, R4 = 30, and R5 = 35) for studying the carbon transformation during composting (Table 3, entry 5). The trends of HA content in these five composting piles increased during the whole composting period. Compared with the beginning of the composting period, the improvement of HA content in R4 and R5 cases was 23.5–33.1% lower than that in R1, R2, and R3 cases. The reason indicated by authors was that the high C/N ratio (>30) causes excessive loss of total organic carbon, which is detrimental to the production and accumulation of HA in the compost pile and has a significant negative impact on the degree of humification of the compost product. They also pointed out that the C/N ratios of 20–25 are more favorable to the HA production in AC. Silva et al. (2014) obtained a similar result that the composting pile with a C/N ratio of 24.1:1 had the highest degree of aromatization and polymerization of organic matter and the highest HA yield after comparing the several piles with different C/N ratios. A probable reason is that microorganisms need to assimilate five parts of carbon and one part of nitrogen to form their own cell body with decomposing organic matters, while absorbing and using one part of carbon. Meanwhile, they need to consume four parts of organic carbon to obtain energy so that the most suitable C/N for microorganism activity is around 25 (Yin et al., 2019).

4) Additives

The additives including amendments and inoculants are also one of the key parameters for humification processes of biomass waste conversion during AC. It was reported that MnO_2_ can promote HA formation by acting on the Maillard reaction through abiotic pathways (Jokic et al., 2004). Zhang et al. (2020) tested MnO_2_ as an additive for the composting of chicken manure and corn straw because MnO_2_ can accelerate HA formation by acting on the Maillard reaction. Compared with the control group (CK), the FA content in MnO_2_ treatment was lower during the whole composting period but the HA yield was higher, as shown in Table 3 (entry 6), suggesting that MnO_2_ can promote the conversion of FA into HA. Although this kind of inorganic chemical additives is beneficial for composting pile maturity, the separation and disposal at the end of composting need to be considered. Common minerals, such as medical stone, another typical kind of inorganic additives, can be applied in composting because it can improve the porosity of the composting pile. Some researchers tested the performance of medical stone in composting, aiming at improving the degradation and humification of pig manure (Wang Q. et al., 2017). As a result, the increase of medical stone addition led to degradation of the FA content. At the composting time of 60 days, the HA content was monitored at 15.13, 16.36, 16.95, 17.90, and 18.81%, while increasing the MS amount from 2.5% to 10%. It was found that this kind of additive is also good for the humification reaction. Compared with the aforementioned chemical additives, minerals are much more environmental-friendly, easier to dispose, and have higher cost-saving benefits. Adenosine triphosphate (ATP) and malonic acid (MA) as organic additives can effectively inhibit CO_2_ emission during AC, which is beneficial for conversion of organic carbon into HSs simultaneously (Lu et al., 2018; Yin et al., 2015). Wang et al. (2019) tested ATP and MA as additives for the composting of chicken manure and garden waste, and the specific composting condition of their study is presented in Table 3 (entry 7). The results were that HA yield first decreased and then increased during the whole composting period, and the HA yields in ATP and MA treatments were higher than those in the CK group, indicating that the addition of ATP and MA promotes the HA formation. As compared to inorganic additives, organic additive utilization needs to concern the initial C/N ratio of composting pile and the influence of this new carbon source on microbial activity during composting. Different organic additives used in composting will lead to different mechanisms of microbial activity. The possible pathways of HS formation during composting need to be greatly concerned. The effects of another kind of additives (i.e., inoculants) on humification in composting have also been investigated by researchers. Chen et al. (2019) found that inoculated *P. chrysosporium* can promote lignin degradation wherein the composting feedstock compared on composting day 0 and day 18 (Chen et al., 2019). The important conditions of the experiments are shown in Table 3 (entry 9). The result indicated by the authors was that the HA content of S2 treatment increased rapidly from day 18 when comparing with that of S1 and S3, and it was significantly higher than that of S1 and S3 at the end of composting, reaching 94.97 g kg^−1^. A plausible reason was also given that the cooling period is beneficial for lignin degradation according to *P. chrysosporium* change. The degradation products are the key precursors of HA formation. Similar results were reported by other studies. For example, Zeng et al. (2009) used a *P. chrysosporium* inoculant for investigating its effect on composting maturity, as presented in Table 3 (entry 10). The highest humification ratio of 51.25 in Run 3 was obtained when comparing with Run 1 and Run 2, indicating that the inoculation period had a significant effect on humification. Thus, there is a need of considering the timing of addition for inoculants because their activity is significantly affected by the composting environment.

5) Aeration intensity

Oxygen directly determines the activity of aerobic microorganisms; thus, it affects the amount of HS production. It is generally considered that a pile of oxygen concentration of 50–150 ml L^−1^ is more suitable for composting. Too low oxygen concentration will lead to an anaerobic environment, while too high oxygen concentration will make microbial activity too vigorous and organic matter decomposition too fast, leading to the reduction of HS production. Xu et al. (2021) investigated the effect of aeration intensity (0.24, 0.36, and 0.48 L kg^−1^ DM min^−1^) on humification of kitchen waste during composting. The best degree of humification was achieved at an aeration intensity of 0.24 L kg^−1^ DM min^−1^, ascribing that low aeration intensity caused slow mineralization of organic substances to promote HS precursor formation. Wu et al. (2019) also reported the effects of aeration rates on the degree of humification *via* composting. At the end of the composting period, the HS concentration was increased by 21.1, 26.4, and 22.4%, where the aeration rates were 0.05, 0.1, and 0.15 L kg^−1^ DM min^−1^ when comparing with the initial HS content. Therefore, the effect of aeration intensity on the humification degree of biomass waste during composting is significant. It is worth optimizing aeration intensity in composting processes.

6) Pretreatment method

In order to promote biomass waste biodegradation and HS conversation during composting, a suitable pretreatment for raw materials is needed. Zhu et al., 2021 pretreated dairy manure at 90°C for 4 h and then placed it into the composting reactor. The conditions of composting are shown in Table 3 (entry 13). The results indicated that thermal pretreatment significantly enhanced the humification degree of AC. Luan et al. (2020) studied the effect of the pretreatment method of composting on biomass (i.e., chicken manure and straw) humification, in which one-time composting fermentation and continuous composting fermentation (pretreatment in the fermenter at high temperature for 3 h) were conducted. The result was obtained that the HS (HA) yield increased from 3.3% to 32.1% (1.7%–56.1%) under the secondary composting fermentation. This is because thermal pretreatment can partly convert the compounds (e.g., cellulose and lignin) into small organic molecules (monosaccharides and phenols, etc.) followed by generating further conversion under microbial reactions.

In summary, although some literatures on revealing the effects of the influencing factors on the humification degree in AC have been carried out, there is still a lack of systematic research works. The carbon transform behavior is worth being tracked and studied during AC, in order to improve the carbon selectivity in the formation of HSs. The economic analysis of composting after optimizing these parameters should be performed, reflecting the potential application of the processes in industry.

### 3.3 Hydrothermal Humification for Biomass Waste Conversion

HT of biomass waste is another way to generate FA, humins, and HA. FA is difficult to extract because of its acid–base solubility and usually exists in the liquid phase together with other soluble organic compounds during the hydrothermal reaction. It is usually qualitatively characterized by three-dimensional fluorescence spectrum, FT-IR spectroscopy, and other detection methods (Usman et al., 2019). Therefore, it is not particularly concerned in this review. For humins, despite their advantage of easy separation due to their characteristic of acid–base insolubility at room temperature, the solid residue obtained after HT of biomass waste (so-called hydrochar) still contains a portion of raw material residue as well as other components that have not yet been decomposed. Therefore, hydrochar cannot be fully regarded as a humus-like material. Recently, studies mainly focused on reaction systems that used homogeneous substances such as glucose and fructose as raw materials to prepare the humins *via* hydrothermal technology (van Zandvoort et al., 2013; Hoang et al., 2015b; Korner et al., 2019; Shi et al., 2019; Xu et al., 2020). Hydrothermal technology can also be used as an extraction method to obtain HA from humus-rich biomass such as coal process waste and lignite (Cheng et al., 2019; Sriramoju et al., 2020). The research studies on direct hydrothermal humification of biomass waste have also appeared (Yang et al., 2019). In this section, a review of hydrothermal humification of biomass waste and their single components in recent years is presented, with special attention to the influence of some important parameters on the degree of humification.

#### 3.3.1 Effect on Humin Formation

Humins are usually regarded as by-products formed during the hydrothermal reaction because of their relative low value compared with other value-added chemicals such as furans and levulinic acid (Jung et al., 2021; Kumar et al., 2020; Sajid et al., 2020; Shao et al., 2020). Although the humin formation is adverse in biorefinery, it is inevitable due to its low activation energy. For instance, Weiqi & Shubin (2017) indicated the activation energy for conversion of glucose to humins (86 kJ/mol) using phosphoric acid and chromic chloride as catalysts in the hydrothermal system, close to the activation energy for glucose conversion to 5-hydroxymethylfurfural (HMF) (65 kJ/mol). Sajid et al. (2020) reported the lower activation energy (24.94 kJ/mol) for fructose conversion to humins comparing with that of conversion of fructose to HMF (33.75 kJ/mol) in a p-toluene sulfonic catalyzed system, and 78.39 kJ/mol for humin formation as well as 96.51 kJ/mol for HMF production in an oxalic acid–catalyzed system. It was reported that the selectivity of humins from carbohydrates can be up to more than 50% in some catalytic systems (Hu et al., 2012; Ordomsky et al., 2013; Weingarten et al., 2011). Since humins are inevitably and massively formed during hydrothermal reactions, they can also be used as a valuable material. Some published literatures highlighted the application of humins such as preparation of hydrogen (Hoang et al., 2013), value-added chemical and biofuel production (Hoang et al., 2015a; Wang Y. et al., 2017; Wang et al., 2016), and catalyst synthesis (Yang J. et al., 2020). Hoang et al. (2015a) summarized the studies on value-added chemicals such as HMF and FF synthesis from (poly)saccharides under HT, accompanied by the humin formation (Hoang et al., 2015a). In their hydrothermal case, the considerable humin yield (35 wt%) derived from glucose was achieved (Table 4), which was then used as a carbonaceous source for synthesis gas production. They also concluded that the humin selectivity depends on reaction temperature, substrate types, catalysts used, etc. Similarly, Kang et al. (2018) reported the values of humin yield associated with various hydrothermal conditions such as different reaction temperature, substance types, and catalysts. However, the detailed effects of these parameters on humin formation need to be analyzed and summarized.

1) Reaction temperature

**TABLE 4:** Effects of various parameters on the degree of humification during the hydrothermal reaction.

Entry	Substance	Variable		Other condition	Degree of humification	Reference
1	Glucose (90 g)<	—		Solvent: 500 ml water; 0.01 M sulfuric acid; reaction temperature: 180°C; reaction time: 6 h	The humin yield of 35 wt% as a carbonaceous source for synthesis gas production	Hoang et al. (2015b)
2	Sugarcane exocarp	—		Reaction temperature: 200°C; reaction time: 1 h; pressure: 15 atm	Sugarcane exocarp nutrient solution contained 17% of humic acid as a liquid organic soil conditioner that can promote crop growth when applied to crop foliar fertilizers	Chang & Huang, (2018)
3	Glucose (2 M)	Reaction temperature (°C)	113	Solvent: water; catalyst: 0.055 M sulfuric acid; reaction time: 6 h	The humin yield was between 3 and 34 wt% improving with the increase in reaction temperature	Van Zandvoort et al. (2013)
			180			
			247			
4	Glucose (5 g)	Reaction temperature (°C)	100	Solvent: [BMIM]Cl; catalyst: CrCl_3_; reaction time: 4 h	The solid humin yield increased from 29.7% to 78.7% as the reaction temperature increased	Xu et al. (2020)
			110			
			120			
			130			
5	Lignite (20 g)	Reaction temperature (°C)	130	Alkali-to-carbon mass ratio of 1:1; water-to-carbon mass ratio of 1: 20; reaction time: 7 h	The HA yield increased sharply from 20% to 90.2% as the temperature increased from 130 to 190°C and then increased slowly until the temperature rose to 210°C	Cheng et al. (2019)
			150			
			170			
			190			
			210			
6	Coal processing waste	Reaction temperature (°C)	130	KOH concentration of 5%	The HA yield increased to 18.8% till the reaction temperature of 140°C; thereafter, the change was not significant	Sriramoju et al. (2020)
			140			
			150			
			160			
			170			
7	Broccoli waste (150 g)	Reaction temperature (°C)	184	Solvent: 100 g water; reaction time: 10 min	The humification rate increased from 0.16 to 0.25 as the reaction temperature increased	Sui et al. (2021)
			204			
			220			
8	Cabbage leaf (3 g)	Reaction temperature (°C)	155	KOH concentration: 25%, NH_4_OH concentration: 10%; reaction time: 1 h	The humic acid yield increased first and then decreased; the highest yield of 0.61% was achieved at a reaction temperature of 195°C	Wang et al. (2017a)
			175			
			195			
			215			
			235			
9	Food waste	Reaction temperature (°C)	175	Pressure: 4.0 MPa; reaction time: 50 min	The humic substance content in the solid phase increased to 39.52% at a reaction temperature of 205°C and then slightly decreased with the increase in temperature	Shu et al. (2016)
			190			
			205			
			215			
10	Carbohydrates (0.12 mol C)	Raw material type	Glucose	Solvent: 30 ml water; reaction temperature: 220°C; reaction time: 5 h	Different humin yields of 35.2–60.7% were found	Shi et al. (2019)
			Fructose			
			Rhamnose			
			Xylose			
11	Broccoli waste (150 g)	Reaction time (min)	10	Solvent: 100 g water; reaction temperature: 204°C	The humification rate increased from 0.19 to 0.22 as the reaction time increased	Sui et al. (2021)
			20			
			40			
12	Lignite (20 g)	Reaction time (h)	3	Alkali-to-carbon mass ratio of 1:1; water-to-coal mass ratio of 1:20; reaction temperature: 190°C	The HA yield increased from 54.4 to 90.2% as the reaction time varied from 3 to 7 h; then, the HA yield decreased from 90.2% to 84.7% with the increase in reaction time	Cheng et al. (2019)
			5			
			7			
			9			
			11			
13	Cabbage leaf (3 g)	Reaction time (h)	1	Reaction temperature: 195°C; KOH concentration: 25%, NH_4_OH concentration: 20%	The humic acid yield increased first and then decreased; the highest yield of 0.76% was achieved at a reaction time of 4 h	Wang et al. (2017a)
			2			
			3			
			4			
			5			
			6			
14	Sewage sludge (300 g, 92% moisture content)	Reaction time (h)	1	Reaction temperature: 200°C	The humin yield was between 15 and 16 g/L	Malhotra & Garg, (2020)
			3			
			5			
			8			
15	Fructose (100 g/L)	pH	2.2	Different ratios of 2 M K_2_HPO_4_ solution and 1 M citric acid; reaction temperature: 140°C	The humin yield was between 1.4 and 64.8%, decreasing with the increase in pH	Korner et al. (2019)
			3			
			4			
			5			
			6			
			7			
			8			
16	Lignite (20 g)	Alkali-to-carbon mass ratio	0.3	Reaction temperature: 190°C; water-to-carbon mass ratio of 1: 20; reaction time: 7 h	The HA yield increased rapidly from 35.5% to 80.8% and then remained stable at 90.7%	Cheng et al. (2019)
			0.5			
			0.7			
			1.0			
			1.5			
17	Coal processing waste	KOH concentration (%)	3.5	Reaction temperature: 140°C	The yield of humic acid (about 12–19%) was increased significantly till 4.5% KOH concentration; after that, the change was not significant	Sriramoju et al. (2020)
			4.0			
			4.5			
			5.0			
			5.5			
			6.0			
18	Cabbage leaf (3 g)	KOH concentration (%)	5	Reaction temperature: 195°C; NH_4_OH concentration: 10%; reaction time: 1 h	The humic acid yield increased rapidly with the increase in KOH concentration till the KOH concentration was 25% (5 times the initial value); then, the change was not significant	Wang et al. (2017a)
			15			
			25			
			35			
			45			
		NH_4_OH concentration (%)	5	Reaction temperature: 195°C; KOH concentration: 25%; reaction time: 1 h	The humic acid yield improved with the increase in NH_4_OH concentration, but the yield was between 60% and 77%, not significantly changed	
			10			
			15			
			20			
			25			
19	Waste biomass (1.2 g)	Raw material type	Glucose	KOH; reaction temperature: 200°C; reaction time: 24 h	The humic acid yield of glucose, sawdust, and tulip trees was 2.3, 1.2, and 1.8%, respectively	Yang et al. (2019)
			Corewood sawdust of beech			
			Tulip trees			

Reaction temperature is an important factor for humin formation, and this part summarizes the relationship between the reaction temperature and humification degree, shown in Table 4. van Zandvoort et al. (2013) investigated the effect of reaction temperature on humins from glucose as the hydrothermal time was fixed at 6 h and using 0.055 M sulfuric acid as a catalyst. The obtained humin yield increased from 3 wt% to 34 wt% with the increase of reaction temperature (entry 3, Table 4). They also built the surface response model and equation that can reflect the humin yield changing with the parameter of reaction temperature. Xu et al. (2020) got a similar result that the solid humin yield improved from 29.7% to 78.7% as the reaction temperature was increased in their hydrothermal system, as shown in Table 4 (entry 4). Therefore, it can be concluded that the improvement of reaction temperature helps to increase humin formation during hydrothermal reactions because higher energy input impels more complex reactions to occur which is conducive to form the humins.

2) Raw material type

The types of raw material also play a vital role in humin formation during the hydrothermal process. Shi et al. (2019) selected glucose, fructose, rhamnose, and xylose as typical raw materials for solid humin production, whose reaction condition is shown in Table 4 (entry 10). The yields of humins based on glucose, fructose, rhamnose, and xylose were 60.7, 54.1, 35.2, and 46.4%, respectively, indicating that the raw material type greatly impacts the humin formation. The higher humin yields achieved from glucose and fructose may ascribe to the HMF formation during hydrothermal reactions, which has the highest humification rate (64.6%). The humin yields from rhamnose and xylose were higher than those from 5-methylfurfural and furfural (FF), indicating that the formation of humins from rhamnose and xylose may be through other routes in addition to 5-methylfurfural and furfural. Selecting a suitable raw material is important for humin formation, and the pathway mechanism of humification of various raw materials is worth studying further. It is suggested to build a database that presents the humin formation pathway based on each kind of raw biomass material and also provides the typical humin structure from various feedstocks.

3) pH value

The pH value in hydrothermal reactions can significantly affect the humin yield as well. Korner et al. (2019) investigated the influence of different pH values (2.2–8), which were adjusted by different ratios of 2 M dipotassium hydrogen phosphate solution and 1 M citric acid on fructose-derived humin yield. The result was that the humin yield decreased from 64.8% to 1.4% while the pH increased, revealing that the humins are soluble in alkaline hydrothermal circumstances. Hoang et al. (2015b) also obtained the same phenomenon that the dissolved fraction of humins was 15.8 wt% higher in NaOH solution than that in water and in acetone, which is the reason for ascribing to partial conversion of humins into alkaline soluble HA. Kang et al. (2016) used glucose-derived humins as raw materials for alkaline catalytic HT. The solid humins completely dissolved as black homogeneous solution after the alkaline hydrothermal reaction and the precipitate known as HA formed after acidification of this solution. The yield of HA was up to 67 wt%, indicating that the conversion of humins to HA in alkaline hydrothermal circumstances is feasible. Similarly, van Zandvoort et al. (2015) tested three types of humins that were derived from glucose, fructose, and xylose for alkaline HT and obtained 75 wt% HA yields. Therefore, different pH conditions in hydrothermal circumstances can make the FA, HA, and humins interchangeable.

#### 3.3.2 Effect on the Formation of Humic Acid

HA is a typical kind of HS that can be easily prepared from various biomass wastes *via* hydrothermal technology (Shu et al., 2016; Wang J. et al., 2017; Chang & Huang, 2018; Yang et al., 2019; Malhotra & Garg, 2020; Sriramoju et al., 2020; Sui et al., 2021). Chang & Huang (2018) produced a value-added nutrient solution containing 17% of HA in HT by using sugarcane exocarp as the raw material. This product was used as a liquid organic soil conditioner that can promote crop growth. This example indicates a great application potential for the hydrothermal humification process that converts biomass waste into HA. The first work that reviews recent literatures on HA production from biomass waste *via* hydrothermal process was carried out in this section. The effect of various parameters on HA production was summarized like humins, as shown in Table 4.

1) Reaction temperature

Lignite and coal, which are typical mineral resources suffering long-term physicochemical reactions, contain abundant HA, which can be readily extracted by hydrothermal technology. The reaction temperature is one of the most important parameters that greatly affect the HA yield extracted from these mineral resources. Cheng et al. (2019) used lignite as the raw material to investigate the effect of reaction temperature on HA yield at a reaction time of 7 h in alkaline hydrothermal circumstances. The HA yield increased sharply from 20% to 90.2% as the temperature increased from 130 to 190°C and then increased slowly until the temperature rose to 210°C. Sriramoju et al. (2020) obtained a similar result that the HA yield improved with the increase in reaction temperature while using coal processing waste as raw materials under alkaline hydrothermal conditions. As for other types of biomass waste, similar phenomena can be observed. For instance, Sui et al. (2021) used broccoli waste as the raw stock for hydrothermal humification to investigate the influence of the reaction temperature on the degree of humification. The humification rate calculated by the content of FA and HA increased from 0.16 to 0.25 as the reaction temperature increased from 184 to 220°C. Raising the reaction temperature in a certain degree is beneficial for HA extraction and production. However, too high reaction temperatures also go against the HA formation. Wang J. et al. (2017) presented the result that the HA yield based on cabbage leaf increased first and then decreased when the reaction temperature increased from 155 to 235°C. The highest yield of 0.61% was achieved at a reaction temperature of 195°C. This is because HA is unstable in hydrothermal environments at a high temperature with ongoing degradation and transformation. The same phenomenon can be obtained in another study that used more complex food waste as raw materials for hydrothermal humification (Shu et al., 2016). In their trials, the HS content in solid products (hydrochar) was detected by a traditional HS extraction method, and the HS yield increased to 39.52% at a reaction temperature of 205°C and then slightly decreased with the further increase in temperature. Therefore, the hydrothermal temperature for HS production needs to be optimized in order to find the best point of degree of humification.

2) Reaction time

Reaction time is another key parameter for the conversion of biomass waste into HA. Sui et al. (2021) reported that the humification rate increased from 0.19 to 0.22 as the reaction time increased when using broccoli waste as a feedstock (entry 11, Table 4). Similar to the reaction temperature, it needs to control the reaction time for a good yield of HA because excessive energy input will lead to degradation and transformation of HA. For example, Cheng et al. (2019) fixed the reaction temperature, alkali-to-carbon mass ratio, and water-to-coal mass ratio at 190°C, 1:1, and 1:20, respectively, to investigate the changes of HA yield affected by the reaction time from 3 to 11 h. The HA yield derived from lignite first increased from 54.4 to 90.2% as the reaction time varied from 3 to 7 h and then decreased with the increase in reaction time. Wang J. et al. (2017) tested the cabbage leaf for hydrothermal humification and obtained the same result as shown in Table 4 (entry 13).

Specifically, sludge as another typical raw material with more than 90% moisture content can also conduct the hydrothermal humification test. This process not only solves the problem of sludge disposal but also transforms the waste into useful and valuable products in line with the concept of sustainability. Malhotra & Garg (2020) investigated the effect of reaction time (1–8 h) on humin generation based on sewage sludge in HT. The humin yield between 15 and 16 g/L could be achieved, presenting good potential for sludge hydrothermal humification. In addition, the solid product so-called hydrochar could also be utilized, indicating the superiority of the hydrothermal humification process for sludge.

3) Alkaline hydrothermal condition

The content of alkali addition can play a key role in HA production as summarized in Table 4 (entry 16–18). Cheng et al. (2019) increased the alkali-to-carbon mass ratio from 0.3 to 1.5; the corresponding HA yield from lignite increased rapidly from 35.5% to 80.8% and then remained stable at 90.7%, ascribing to the addition of KOH that broke the chelation between HA and metal ions. Sriramoju et al. (2020) also reported a similar result that the yield of HA from coal processing waste (about 12–19%) was increased significantly till 4.5% KOH concentration; after that, the change was not significant. These results indicate that the amount of alkali was positively correlated with the extraction and formation rate of HA. However, the cost of alkali addition needs to be considered, and the more the alkali addition, the more the acid addition in subsequent HA separation.

Various kinds of alkali also have different influence on HA preparation under hydrothermal reactions. Wang J. et al. (2017) tried two kinds of alkali (i.e., KOH and NH_4_OH) as additives for cabbage leaf humification during the hydrothermal process. A trend for changes of HA yield with the content of KOH addition was observed. This was because the addition of alkali in the hydrothermal reaction can serve to delignify lignin, which is an important precursor for HA generation. On the other hand, HA is a weak acid, and the acidic group [H^+^] of HA can be replaced by a metal ion [K^+^] to produce soluble HA. The increase of NH_4_OH concentration could not significantly affect the HA yield, indicating that the strong alkali has a better effect on HA production than the weak alkali. Therefore, the selection of alkali is important for HA production.

Alkaline hydrothermal condition can directly convert biomass waste into HA. Yang et al. (2019) first tried the process of hydrothermal humification of biomass waste under alkaline circumstances. They tested three kinds of raw material (i.e*.*, glucose, wood sawdust of beech, and tulip trees), and achieved an HA yield of 2.3, 1.2, and 1.8%, respectively. Comparing with other humification process of biomass waste, the HA yields achieved were significant lower. Shao et al. (2022) developed the hydrothermal process that combined the acid hydrothermal pretreatment for biomass waste and alkaline hydrothermal humification, aiming to further improve the HA yield. The highest HA yield based on biomass waste (dry basis) achieved until now is 28.74 wt%. The field of hydrothermal humification of biomass is emerging, and this is a chance for researchers to focus on it.

## 4 Composting Integrated With Hydrothermal Treatment

After separately introducing the mechanisms of the humification processes (i.e., AC and HT) and summarizing the effects of various parameters on the humification degree, the work of comparison of these two processes was carried out to further reveal their characteristics.

**FIGURE 4:**
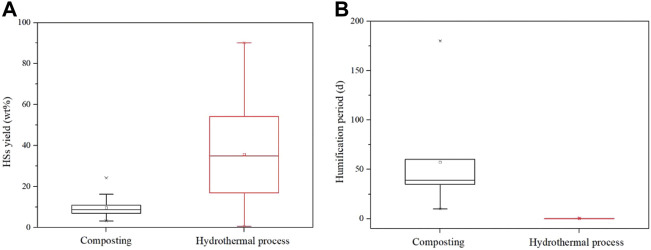
Comparison of composting and hydrothermal treatment in **(A)** HS yield and **(B)** humification period (the data are referred from the literatures listed in Tables 3, 4).

The humification degree by using AC or HT is the target indicator in this review. Therefore, the comparison of these humification processes in the HS yield was conducted as shown in Figure 4A. The HS yield based on AC is almost below 20 wt%, concentrated at approximately 10 wt%, and the range of the HS yield is also narrow, indicating a similar level of degree of humification from composting, although the composting conditions reported are varied. The HS yields obtained in HT have a large range of values, from 1.2 wt% to 90.2 wt%, depending on the type of the raw materials. Specifically, the HS yield of 1.2 wt% is from a kind of lignocellulosic biomass waste sawdust, and the highest HS yield is extracted from lignite. Also, the median value of the HS yield is 35.8 wt%, which is higher than that of composting, indicating the great potential of HT for HS production. The humification period of composting and HT is significantly different, as shown in Figure 4B. The composting period summarized is in the range of 10–180 days, and its median value is located at 35 days, which is more than 1 month, while the period in HT is only less than 1 day, indicating the good efficiency of HT for humification processes of biomass waste. In addition, the solvent circumstance created by the HT process is beneficial for HS (HA and FA) dissolution. The HA can be easily extracted and purified from the liquid phase by adjusting the pH of liquid to 1–2 subsequently. However, the hydrothermal process needs lots of energy input because the used reaction temperature is usually over 160 °C, thus improving the cost (Zhou et al., 2021). In addition, the disposal scale of the hydrothermal process still stays in the laboratory, with the expectation that it will approach the level of composting. In spite of this shortcoming, the productivity of HSs in AC and HT is recommended to be calculated based on the consideration of the HS yield, reaction time, cost of additives, and energy input. Until now, the correlated study is insufficient and sought.

## 5 Combination of Hydrothermal Treatment With Composting

**FIGURE 5:**
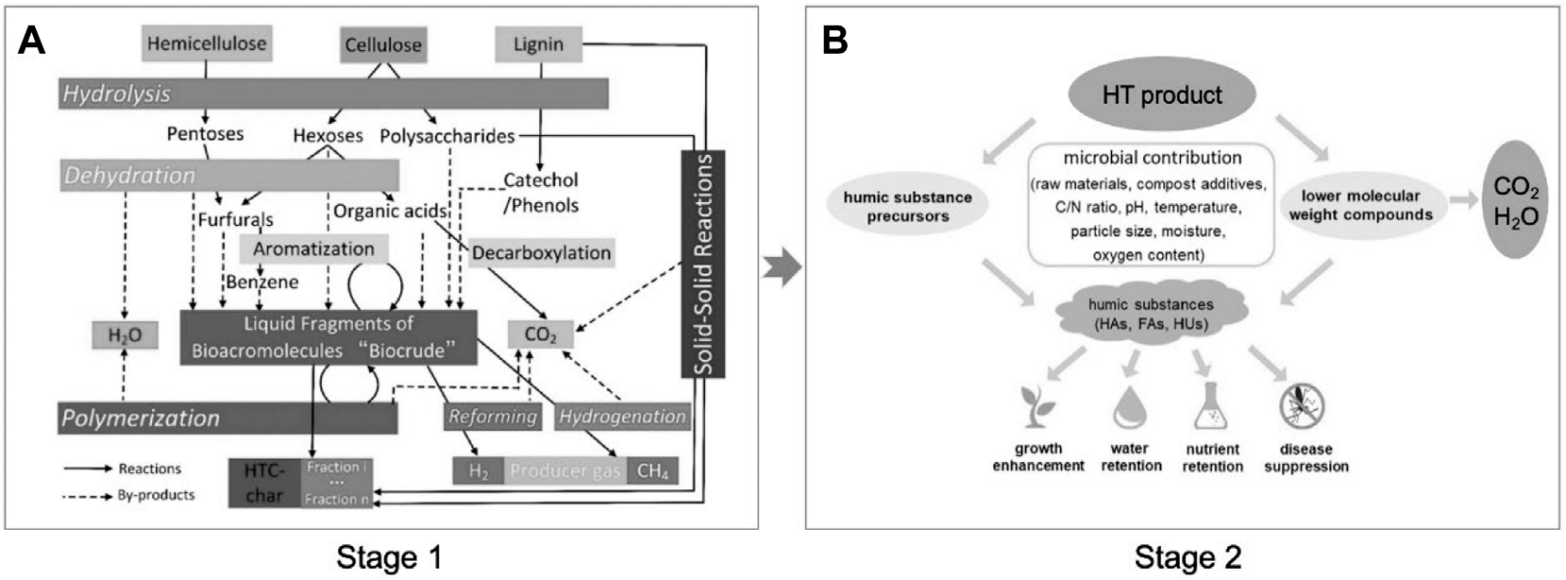
Schematic diagram of the combination process of hydrothermal treatment (stage 1) with composting (stage 2). **(A)** Reproduced from the work by Kruse et al. (2013) with permission from Elsevier, copyright 2013; **(B)** reproduced from the work by Guo et al. (2019) with permission from Elsevier, copyright 2019.

It needs a long period for humification processes in AC. When HT was used as a pretreatment method integrated with AC, the humification processes can be accelerated efficiently with a rapid degradation of raw biomass (Figure 5). Varied small molecular compounds can be obtained after hydrothermal reactions as shown in stage 1; then, they are used as precursors for further humification in AC. Currently, there are several literatures reported on this system with the combination of HT and AC. Nakhshiniev et al. (2014) evaluated the HT for enhancing composting in terms of stability and maturity of lignocellulosic agricultural residues. The result presented that the time with hydrothermal pretreatment needed only 6 weeks for maturity, while the composting period of pile without hydrothermal pretreatment was more than 14 weeks. Gong et al. (2021) used hydrothermal technology to pretreat the oxytetracycline fermentation residue and expected to be effective for later composting. The result indicated that the oxytetracycline fermentation residue and its intermediate content decreased significantly, but the germination index of this compost did not change notably. The more important phenomenon was that the composting period was shortened from 28 to 14 days, proving that the hydrothermal pretreatment is beneficial for humification in AC. It was reported that the composting pile *via* hydrothermal pretreatment may occur due to the toxic furans that can inhibit the activity of composting microorganisms; this problem can be efficiently solved. Nakasaki et al. (2015) used food waste after hydrothermal pretreatment as the compost raw material and obtained the obvious result that the degradation of organic matter was inhibited (Nakasaki et al., 2015). However, they discovered a new fungus (*Paecilomyces* species FA13) having the capacity of furan degradation that can accelerate the composting pile maturity.

In summary, a combination of HT with composting has been proved that can accelerate the biomass waste maturity. Although the inhibition factor existed in AC integrated with HT processes has been concerned, the HS content and its transformation are worth concerning. It is recommended to focus on the relationship between biomass raw materials and transformed intermediate compounds during hydrothermal pretreatment in terms of types, concentration, proportion (stage 1), and HS formation in AC (stage 2). This point can help to further understand about the effect of hydrothermal pretreatment on the humification behavior during AC, which is worth studying.

## 6 Conclusion

In summary, we systematically reviewed the development in artificial humification processes of biomass waste conversion based on aerobic composting (AC) and hydrothermal treatment (HT) in humic substance (HS) production. The HSs involving humic acid, fulvic acid, and humins have great promising application for soil remediation, battery, adsorbents, and so on. This is the first time that a study has particularly briefed the varied influencing factors on the humification degree in AC and HT. Meanwhile, the major aspects of mechanisms, characteristics of HSs’ molecular structure, and element composition in humification processes are summarized and discussed. The practical issues in aerobic composting such as low yield of HSs, long-period reaction, complex by-products, and hard purification are discussed. The hydrothermal treatment has some salient features in the aspect of shortening reaction period, promoting yields of HSs, and wide adaptability of biomass raw materials. However, the strong randomness in HS yields of 1.2–90.2 wt% in hydrothermal humification needs to be addressed prior to application in wide areas. It is suggested that future studies can be extended to investigate (1) hydrothermal humification optimization, (2) combining aerobic composting with hydrothermal treatment, and (3) multi-techniques integrated in high-effective humification processes.
